# Factors associated with health-related quality of life and financial toxicity among gynecological cancer patients in Southern Nigeria

**DOI:** 10.1038/s41598-025-13763-0

**Published:** 2025-07-31

**Authors:** Chibuzor F. Ogamba, Adedayo Joseph, Ayodeji Kayode Adefemi, Chidike Onyedikachi Ezegwui, Ajay Major, Rasaq Oluwagbemiga Jimoh, Michael Osahumen Orhue, Michael Chudi Ezeanochie, Boniface Uji Ago, Linda Ogochukwu Amah, Emeka Emmanuel Okoh, Courage Osamudiamen Idahor, Mazpa Ejikem, Alexander Clive Tinworth, Charilaos Zisou, Nwamaka N. Lasebikan

**Affiliations:** 1 Nuffield Department of Population Health, University of Oxford, Oxford, OX3 7LF UK; 2 NSIA-LUTH Cancer Centre, Lagos University Teaching Hospital, Lagos, Nigeria; 3 Department of Radiation Biology, Radiodiagnosis and Radiotherapy, College of Medicine, University of Lagos, Lagos, Nigeria; 4 Department of Obstetrics and Gynaecology, Lagos State University Teaching Hospital, Ikeja, Lagos State Nigeria; 5 Department of Obstetrics and Gynaecology, University of Nigeria Teaching Hospital, Ituku-Ozalla, Enugu, Enugu State Nigeria; 6 Division of Haematology, Department of Medicine, University of Colorado School of Medicine, Aurora, CO USA; 7 Faculty of Clinical Sciences, College of Medicine, University of Lagos, Lagos, Nigeria; 8 Department of Obstetrics and Gynaecology, University of Benin Teaching Hospital, Benin, Edo State Nigeria; 9 Department of Obstetrics and Gynaecology, University of Calabar Teaching Hospital, , Cross-Rivers State, Calabar, Nigeria; 10 University of Nottingham, Nottingham, UK; 11 Nnamdi Azikiwe University Teaching Hospital, Awka, Nigeria; 12 Nottingham University Hospital, Nottingham, UK; 13 Department of Anaesthesiology, SUNY Downstate Health Sciences University, New York, USA; 14 Department of Radiation Oncology, University of Nigeria Teaching Hospital, Ituku-Ozalla, Enugu, Enugu State Nigeria

**Keywords:** Quality of life, Financial toxicity, Gynecological cancer, Nigeria, Cancer, Health care, Medical research, Oncology

## Abstract

The health-related quality of life (HRQoL) and financial toxicity (FT) of women with gynecological cancers in sub-Saharan Africa have not been sufficiently explored. This study assessed predictors of HRQoL and FT, as well as the effect of FT on HRQoL, among affected women in Nigeria. This cross-sectional study investigated 574 women with gynecological cancers receiving care at various stages at five academic hospitals in southern Nigeria between June 2022 and September 2024. HRQoL and FT were evaluated using the FACT-G and FACIT-COST tools, respectively. Multivariable linear regression models estimated the associations of patient characteristics with HRQoL and FT and the effect of FT on HRQoL. Most women experienced FT. After multivariable adjustments, unemployed women, women with ovarian and uterine cancers and choriocarcinoma, higher disease stages, and worse ECOG status had worse HRQoL. Conversely, women in remission had better HRQoL. FT was worse with stages III and IV disease and in those on active treatment. However, older women, those with health insurance, higher income, ovarian cancer and choriocarcinoma had better FT. FT varied linearly with HRQoL, with better HRQoL per unit better FT. This effect was stronger in those with a first tumor, those in pre-treatment and those in remission. Our findings identify possible predictors of HRQoL and FT and suggest potential benefits of reducing FT on the HRQoL of women with gynecological cancers in Nigeria.

**Supplementary Information:**

The online version contains supplementary material available at 10.1038/s41598-025-13763-0.

## Introduction

Cancer remains a significant cause of morbidity and mortality globally, with the global burden projected to double over the next two decades, disproportionately affecting low and middle-income countries^1^. Gynecological cancer cases, in particular, account for approximately 15.3% of incident cancer cases and 15.8% of new cancer deaths in women globally^2^. In Nigeria and Africa, gynecological cancers rank among the top five most common cancers overall and in women^3^.

The impact of cancer-related morbidity and treatment on the health-related quality of life (HRQoL) of affected patients, as well as its predictive value on survival outcomes, has been a subject of research interest in recent decades, with an increasing focus on objectively quantifying the subjective experiences of patients using patient-reported outcome measures^4^. In addition to the negative physical and psychological effects of cancer on the quality of life of patients, gynecological cancers also affect the psychosocial and psychosexual functioning of affected women^2,4^. Studies of HRQoL among patients with gynecological cancers in various patient populations have demonstrated associations between demographic and disease-related characteristics such as age, cancer type, disease stage and treatment type on HRQoL, as well as the utility of HRQoL measures across the treatment continuum in predicting survival outcomes^5–8^. These have highlighted the potential benefit of integrating these assessments in clinical trials and management guidelines to identify patients at risk of poor outcomes and evaluate the suitability of various treatments in different patient sub-populations^4,7,8^.

In addition, cancer diagnosis and treatment come with a direct significant financial burden as well as indirect costs associated with reduced productivity and possible loss of employment due to cancer-related morbidity^9^. Financial toxicity (FT) in cancer care refers to the patient-level burden and distress associated with cancer-related costs^9,10^. The impact of these costs is particularly worsened in low-resource healthcare systems characterized by significant out-of-pocket expenditures, such as in Nigeria, where out-of-pocket expenditures constitute approximately 76% of current healthcare expenditure and is the predominant source of cancer care financing, with only 3% of the population aged 15–49 having a form of health insurance^10–14^. This potentially contributes to worse quality of life and survival outcomes due to diagnostic and treatment delays and non-adherence^9,10^. Various studies have highlighted possible risk factors for increased FT in different populations, such as younger age, low income, and lack of insurance, and have also demonstrated inverse associations between FT and HRQoL among gynecological cancer patients^9,10,15–18^.

However, there is a paucity of studies assessing the HRQoL and the experience of FT among patients with gynecological cancers in Nigeria. In addition, the impact of FT on HRQoL and how these differ by various patient characteristics and along the cancer care continuum have not been demonstrated in this population. Studies of HRQoL among women with cancer in Nigeria have mostly evaluated breast cancer patients and, to a lesser extent, cervical cancer patients and have been from mostly single institutions, consequently decreasing the generalizability of findings. Furthermore, many of the available studies perform univariate analyses without accounting for potential confounding factors^19^. To bridge this gap, this study aimed to (1) identify factors associated with both HRQoL and FT among women with gynecological cancers seeking care at multiple Nigerian tertiary facilities and (2) assess the effect of FT on HRQoL overall and for different subpopulations of patients and at different stages of care.

## Methods

### Study design and recruitment

**Fig. 1:**
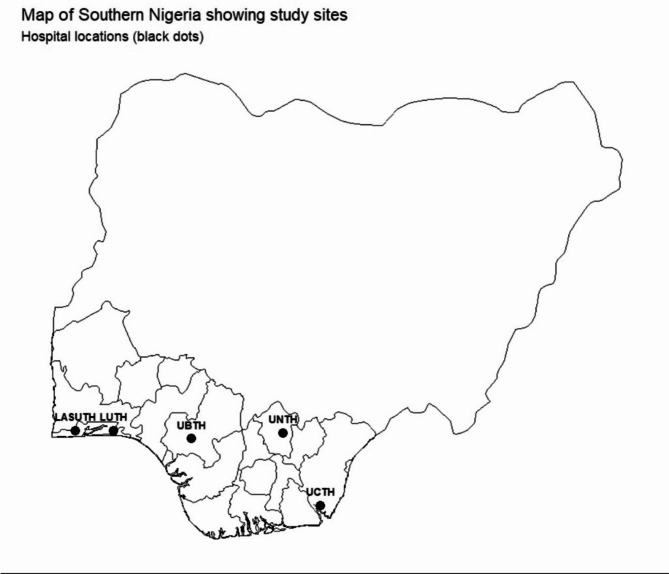
Map of Southern Nigeria showing study locations.

This was a multicenter hospital-based cross-sectional study. The study population consisted of patients with gynecological malignancies seeking care at the specialist outpatient clinics of at least one tertiary hospital in each of the three geopolitical zones in southern Nigeria^20^. The study sites included a total of five tertiary hospitals selected for being major referral centers for cancer care in their respective zones: the Lagos University Teaching Hospital (LUTH, including the NSIA-LUTH Cancer Centre) and the Lagos State University Teaching Hospital (LASUTH) in the southwest region, the University of Nigeria Teaching Hospital (UNTH) Enugu in the southeast, and the University of Benin Teaching Hospital (UBTH) and the University of Calabar Teaching Hospital (UCTH) in the southsouth region (Fig. 1)^21–26^.

Eligible patients receiving care at the above facilities between June 2022 and September 2024, at any point during their disease course—from diagnosis to post-treatment survivorship—were approached by clinicians and research assistants. Women who consented were subsequently recruited. To be eligible for inclusion in the study, participants had to be receiving care for any female reproductive system cancer at any of the centers, at least ambulatory, and capable of self-care. Patients with gynecological cancers who were hospitalized/inpatients or not managed at any of the selected study sites were excluded.

### Ethical considerations

Ethical approvalfor this study was obtained from the Health Research and Ethics Committees (HREC) of all the participating centers, with approval numbers LUTH HREC (ADM/DSCST/HREC/APP/5126), LASUTH HREC (LREC/06/10/1996), UNTH HREC (UNTH/HREC/2022/06/432), UBTH HREC (ADM/E22/A/VOLVII/14831018), UCTH HREC (UCTH/HREC/33/Vol.III/245) and the study was conducted in accordance with relevant guidelines. Informed consent was obtained from all patients prior to their recruitment into the study. Patients were assured of the confidentiality of their information, and no personal details were requested for the study.

### Outcome measures

The primary outcome measures were participants’ HRQoL and FT. HRQoL was measured using the Functional Assessment of Cancer Therapy - General (FACT-G, English language Version 4), a 27-item questionnaire designed to measure four subscales of quality of life: physical well-being (seven items), social well-being (seven items), emotional well-being (six items) and functional well-being (seven items), and which takes 5–10 min to complete^27,28^. The total scores on the questionnaire range from 0 to 108, with higher scores indicating a better quality of life. The FACT-G is a validated HRQoL measure that, since its development, has been translated into 50 languages, has been used worldwide, and has been used in patients with gynecological cancers^27,29^.

FT was assessed by the Functional Assessment of Chronic Illness Therapy Comprehensive Score for Financial Toxicity (FACIT-COST; English language Version 2), a 12-item instrument developed at the University of Chicago and primarily validated in patients with metastatic solid malignancies^18^. It provides a total score ranging from 0 to 44, with lower values indicating greater financial burden. It has been proposed to be graded as no FT (COST scores > 25), mild FT (COST scores 14–25), moderate FT (COST scores 1–13), and severe FT (COST score of 0)^30^. It is one of the few available standardized and well-validated patient-reported outcome measures of FT and has been used previously to study FT in patients with gynecologic malignancies^18,31,32^.

As neither tool has been validated in the Nigerian context, internal consistency reliability was assessed using Cronbach’s alpha, where a raw alpha > 0.70 is generally considered acceptable. The standardized Cronbach’s alpha for the FACT-G was 0.92 (raw alpha: 0.86, 95% CI: 0.84–0.87), and for the FACIT-COST tool, it was 0.87 (raw alpha: 0.75, 95% CI: 0.72–0.78). These values indicate acceptable internal consistency and satisfactory reliability of both instruments in this Nigerian sample.

### Data collection tool

Consenting participants were administered a structured online Google Form questionnaire and printed copies adapted from previous studies, using both self-administered and interviewer-assisted formats^29^. The questionnaires consisted of three sections: patient sociodemographic and clinical characteristics, the FACT-G tool, and the FACIT-COST tool.

Sociodemographic characteristics included age, religion, ethnicity, marital status, employment status, highest level of education, daily household income, health insurance status, type of health insurance, and degree of insurance coverage for cancer care. Clinical characteristics of the patients included diagnosis, Eastern Co-operative Oncology Group (ECOG) performance score, disease stage, disease category (first tumor, relapse, recurrence, second tumor), treatment category (pre-treatment – defined as patients with a diagnosis but who are yet to commence treatments, on active treatment – defined as patients who had undergone treatments and were still receiving treatments, and post-treatment survivorship- defined as patients who had undergone scheduled treatments and were presenting for follow-up care), all types of prior cancer-related treatment undertaken (no treatment, single and combinations of surgery, chemotherapy and/or radiotherapy), and presence of comorbidities. Patient sociodemographic and clinical characteristics were either ascertained from patient medical records or self-reported. Employment status was initially recorded using the following categories: employed, retired, unemployed and job-seeking, unemployed and not job-seeking, and unable to work due to illness or disability. For statistical analysis, these subcategories were collapsed into a binary variable—employed versus unemployed—to preserve statistical power and ensure model stability given the small sample sizes within specific subgroups.

### Statistical analysis

A minimum sample size of 384 was determined using the Cochran formula for sample size calculation, with a 5% margin of error and an estimated proportion of 50% to maximize variability. The minimum sample size was increased by 10% to account for possible contingencies such as non-response.

Histograms and quantile-quantile plots revealed approximately normal distributions in total HRQoL scores and skewed distributions in age, daily household income and total FT scores. The frequency, mean (standard deviation [SD]) and median (interquartile range [IQR]) of participant sociodemographic and clinical characteristics (for categorical, normal continuous and non-normal continuous variables, respectively) were calculated across fifths of total HRQoL score and categories of FT (no [> 25], mild [14-25] and moderate-severe [< 14]), cancer types and HRQoL sub-scales.

Individuals with unfilled FACIT-COST questionnaires were excluded from downstream analyses. Missing data in age, income, disease stage, ECOG score, diagnosis category, and treatment type were addressed using single regression imputation (**Appendix S1**).

Univariable linear regression models were used to estimate the associations of individual sociodemographic and clinical characteristics with overall HRQoL and FT (both continuous), as well as the effect of FT on overall HRQoL. Variables showing univariable associations with *p* < 0.05 were subsequently included in age-adjusted models to determine predictors of HRQoL and FT, respectively. For all categorical variables, the *p*-value from the F-test was used to assess significance. The multivariable model of HRQoL was adjusted for age (continuous), center, religion (Christianity, Islam, other), ethnicity (Yoruba, Igbo, Other), marital status (married, single, widowed/separated/divorced), employment status (employed, unemployed), diagnosis (cervical, ovarian, uterine, vulvar, choriocarcinoma), stage (I to IV), ECOG performance score, diagnosis category (first tumor, relapse/recurrence, second tumor), treatment category (pre-treatment, on active treatment, post-treatment survivorship), and presence of comorbidities (no, yes). The multivariable model of FT was adjusted for age, center, religion, ethnicity, level of education, income (continuous), insurance status (no, yes), diagnosis, stage, diagnosis category, and treatment category. Subsequently, a separate multivariable model was used to estimate the independent effect of FT (continuous) on HRQoL after adjusting for potential confounding effects of variables that showed significant univariable associations (*p* < 0.05) with both HRQoL and FT, namely age, center, religion, ethnicity, diagnosis, stage, diagnosis category, and treatment category. To assess the shape of the association, the adjusted model was evaluated with FT categorized into four groups as above (from no FT to severe FT). Departure from linearity in the association between FT and HRQoL was tested using an F-test, comparing nested models of HRQoL with FT modelled ordinally and categorically. A shape plot of marginal means and 95% CI of HRQoL from the adjusted model was subsequently plotted across the four categories of FT.

Furthermore, the linear effect of FT on HRQoL was evaluated across subgroups of diagnosis, disease stage, diagnosis category, and treatment category to investigate potential effect modification and statistical heterogeneity was assessed through an F-test comparing nested multivariable models with and without an interaction term (between FT and each of the potential effect modifiers). As an additional analysis, the adjusted effect of treatment type on FT was evaluated in a separate model, excluding treatment category due to multicollinearity. Finally, sensitivity analyses to assess the robustness of our findings were conducted on the data using multiple imputation with five imputed datasets with results pooled according to Rubin’s methods (**Appendix S1**)^33,34^. All models were evaluated for robustness to model assumptions. Statistical significance was set at *p* < 0.05. Data were analyzed and plotted in R version 4.3.1 using the following packages: psych, mice, performance, sf, rnaturalearth, rnaturalearthdata, ckbplotr^33,35–37^.

The findings were reported in accordance with the Strengthening the Reporting of Observational Studies in Epidemiology (STROBE) guidelines^38^. (**Table ****S1**).

## Results

### Patient characteristics

A total of 574 women were enrolled in the study. Of these, individuals with unfilled FACIT-COST questionnaires (*n* = 2, 0.3%) were excluded from regression analyses. Missing data were observed for age (*n* = 57, 9.9%), daily household income (*n* = 184, 32.1%), disease stage (*n* = 65, 11.3%), ECOG performance score (*n* = 84, 14.6%), diagnosis category (*n* = 12, 2.1%), and treatment type (*n* = 5, 0.9%). The median age was 53 years (IQR: 44–63) and varied by cancer type, with women with choriocarcinoma and ovarian cancer tending to be younger (Table 1 & S2). Most of the patients were recruited from LUTH (*n* = 262, 45.6%) and LASUTH (*n* = 132, 23.0%), were Christian (*n* = 465, 81.0%), married (*n* = 392, 68.3%), of Yoruba (*n* = 222, 38.7%) or Igbo (*n* = 195, 34.0%) ethnicity, and had attained either tertiary (*n* = 240, 41.8%) or secondary (*n* = 211, 36.8%) education. Although most patients were employed (*n* = 332, 57.8%), the median reported daily household income was ₦3,333.00, and only 10.8% (*n* = 59) of all patients indicated that they were on any form of health insurance, which in most cases covered their health expenditures only partly (*n* = 45, 76.3%).

**Table 1:** Baseline characteristics of participants, overall and by both health-related quality of life and financial toxicity scores.

Characteristic	Overall	Health-Related Quality of Life Score (*N* = 574)					Financial Toxicity Score (*N* = 572)		
	*N* = 574	14–47 *N* = 115	47–53 *N* = 122	53–59 *N* = 112	59–69 *N* = 110	69–103 *N* = 115	No *N* = 41	Mild *N* = 287	Moderate-Severe *N* = 244
**Age (in years)**,** Median (IQR)**	53 (44–63)	53	52	49	56	54	52	53	53
**Centre**,** n (%)**									
*LUTH*	262 (45.6)	16 (13.9)	57 (46.7)	66 (58.9)	76 (69.1)	47 (40.9)	26 (63.4)	168 (58.5)	68 (27.9)
*LASUTH*	132 (23.0)	53 (46.1)	33 (27.0)	22 (19.6)	14 (12.7)	10 (8.7)	4 (9.8)	74 (25.8)	54 (22.1)
*UNTH*	105 (18.3)	22 (19.1)	19 (15.6)	12 (10.7)	16 (14.5)	36 (31.3)	7 (17.1)	21 (7.3)	77 (31.6)
*UBTH*	54 (9.4)	17 (14.8)	9 (7.4)	9 (8.0)	2 (1.8)	17 (14.8)	3 (7.3)	16 (5.6)	33 (13.5)
*UCTH*	21 (3.7)	7 (6.1)	4 (3.3)	3 (2.7)	2 (1.8)	5 (4.3)	1 (2.4)	8 (2.8)	12 (4.9)
**Religion**,** n (%)**									
*Christianity*	465 (81.0)	87 (75.7)	93 (76.2)	90 (80.4)	97 (88.2)	98 (85.2)	29 (70.7)	223 (77.7)	211 (86.5)
*Islam*	100 (17.4)	23 (20.0)	28 (23.0)	20 (17.9)	12 (10.9)	17 (14.8)	12 (29.3)	60 (20.9)	28 (11.5)
*Other*	9 (1.6)	5 (4.3)	1 (0.8)	2 (1.8)	1 (0.9)	0 (0.0)	0 (0.0)	4 (1.4)	5 (2.0)
**Ethnicity**,** n (%)**									
*Yoruba*	222 (38.7)	46 (40.0)	48 (39.3)	55 (49.1)	45 (40.9)	28 (24.3)	18 (43.9)	134 (46.7)	70 (28.7)
*Igbo*	195 (34.0)	38 (33.0)	42 (34.4)	23 (20.5)	40 (36.4)	52 (45.2)	14 (34.1)	84 (29.3)	96 (39.3)
*Hausa*	25 (4.4)	8 (7.0)	7 (5.7)	5 (4.5)	2 (1.8)	3 (2.6)	3 (7.3)	11 (3.8)	11 (4.5)
*Other/missing*	132 (23.0)	23 (20.0)	25 (20.5)	29 (25.9)	23 (20.9)	32 (27.8)	6 (14.6)	58 (20.2)	67 (27.5)
**Marital status**,** n (%)**									
*Married*	392 (68.3)	64 (55.7)	86 (70.5)	74 (66.1)	84 (76.4)	84 (73.0)	30 (73.2)	196 (68.3)	164 (67.2)
*Single*	48 (8.4)	8 (7.0)	13 (10.7)	13 (11.6)	3 (2.7)	11 (9.6)	1 (2.4)	27 (9.4)	20 (8.2)
*Widowed/Separated/Divorced*	134 (23.3)	43 (37.4)	23 (18.9)	25 (22.3)	23 (20.9)	20 (17.4)	10 (24.4)	64 (22.3)	60 (24.6)
**Level of education**,** n (%)**									
*None*	23 (4.0)	2 (1.7)	5 (4.1)	2 (1.8)	10 (9.1)	4 (3.5)	1 (2.4)	11 (3.8)	11 (4.5)
*Primary*	100 (17.4)	19 (16.5)	25 (20.5)	15 (13.4)	22 (20.0)	19 (16.5)	7 (17.1)	39 (13.6)	54 (22.1)
*Secondary*	211 (36.8)	46 (40.0)	40 (32.8)	41 (36.6)	42 (38.2)	42 (36.5)	14 (34.1)	106 (36.9)	90 (36.9)
*Tertiary*	240 (41.8)	48 (41.7)	52 (42.6)	54 (48.2)	36 (32.7)	50 (43.5)	19 (46.3)	131 (45.6)	89 (36.5)
**Daily household income (Naira)**,** Median (IQR)**	3,333 (2,000–8,625)	3,000	5,000	4,000	3,000	3,000	6,000	5,000	2,000
**Employment status**,** n (%)**									
*Employed*	332 (57.8)	61 (53.0)	69 (56.6)	67 (59.8)	57 (51.8)	78 (67.8)	27 (65.9)	167 (58.2)	136 (55.7)
*Unemployed*	242 (42.2)	54 (47.0)	53 (43.4)	45 (40.2)	53 (48.2)	37 (32.2)	14 (34.1)	120 (41.8)	108 (44.3)
**On health insurance**,** n (%)**	59 (10.3)	8 (7.0)	13 (10.7)	14 (12.5)	9 (8.2)	15 (13.0)	8 (19.5)	31 (10.8)	19 (7.8)
**Health insurance type**,** n (%)**									
*Government e.g.*,* NHIS*	33 (55.9)	5 (62.5)	5 (38.5)	9 (64.3)	5 (55.6)	9 (60.0)	4 (50.0)	12 (38.7)	17 (89.5)
*Private e.g.*,* company owned*	21 (35.6)	3 (37.5)	6 (46.2)	2 (14.3)	4 (44.4)	6 (40.0)	3 (37.5)	17 (54.8)	0 (0.0)
*Cancer Health Fund*	1 (1.7)	0 (0.0)	0 (0.0)	1 (7.1)	0 (0.0)	0 (0.0)	0 (0.0)	0 (0.0)	1 (5.3)
*Missing*	4 (6.8)	0 (0.0)	2 (15.4)	2 (14.3)	0 (0.0)	0 (0.0)	1 (12.5)	2 (6.5)	1 (5.3)
**Insurance coverage of cancer treatment**,** n (%)**									
*Not at all*	6 (10.2)	0 (0.0)	1 (7.7)	3 (21.4)	0 (0.0)	2 (13.3)	2 (25.0)	1 (3.2)	3 (15.8)
*Yes*,* Partly*	45 (76.3)	7 (87.5)	8 (61.5)	10 (71.4)	8 (88.9)	12 (80.0)	5 (62.5)	24 (77.4)	15 (78.9)
*Yes*,* Fully*	4 (6.8)	0 (0.0)	2 (15.4)	0 (0.0)	1 (11.1)	1 (6.7)	0 (0.0)	4 (12.9)	0 (0.0)
*Missing*	4 (6.8)	1 (12.5)	2 (15.4)	1 (7.1)	0 (0.0)	0 (0.0)	1 (12.5)	2 (6.5)	1 (5.3)
**Diagnosis**,** n (%)**									
*Cervical cancer*	314 (54.7)	59 (51.3)	48 (39.3)	55 (49.1)	75 (68.2)	77 (67.0)	22 (53.7)	143 (49.8)	149 (61.1)
*Ovarian cancer*	114 (19.9)	24 (20.9)	37 (30.3)	22 (19.6)	13 (11.8)	18 (15.7)	6 (14.6)	62 (21.6)	45 (18.4)
*Uterine cancer*	89 (15.5)	21 (18.3)	21 (17.2)	15 (13.4)	17 (15.5)	15 (13.0)	9 (22.0)	49 (17.1)	30 (12.3)
*Vulvar cancer*	42 (7.3)	9 (7.8)	12 (9.8)	13 (11.6)	4 (3.6)	4 (3.5)	4 (9.8)	20 (7.0)	18 (7.4)
*Choriocarcinoma*	15 (2.6)	2 (1.7)	4 (3.3)	7 (6.2)	1 (0.9)	1 (0.9)	0 (0.0)	13 (4.5)	2 (0.8)
**Stage**,** n (%)**									
*Stage 1*	109 (19.0)	15 (13.0)	24 (19.7)	23 (20.5)	15 (13.6)	32 (27.8)	11 (26.8)	57 (19.9)	39 (16.0)
*Stage 2*	192 (33.4)	36 (31.3)	39 (32.0)	40 (35.7)	42 (38.2)	35 (30.4)	13 (31.7)	110 (38.3)	69 (28.3)
*Stage 3*	144 (25.1)	30 (26.1)	29 (23.8)	26 (23.2)	31 (28.2)	28 (24.3)	9 (22.0)	71 (24.7)	64 (26.2)
*Stage 4*	64 (11.1)	15 (13.0)	14 (11.5)	13 (11.6)	14 (12.7)	8 (7.0)	2 (4.9)	19 (6.6)	43 (17.6)
*Missing*	65 (11.3)	19 (16.5)	16 (13.1)	10 (8.9)	8 (7.3)	12 (10.4)	6 (14.6)	30 (10.5)	29 (11.9)
**Category of diagnosis**,** n (%)**									
*First tumor*	457 (79.6)	77 (67.0)	81 (66.4)	87 (77.7)	102 (92.7)	110 (95.7)	28 (68.3)	220 (76.7)	208 (85.2)
*Relapse/Recurrence*	54 (9.4)	17 (14.8)	17 (13.9)	12 (10.7)	4 (3.6)	4 (3.5)	4 (9.8)	31 (10.8)	19 (7.8)
*Second tumor*	51 (8.9)	14 (12.2)	21 (17.2)	12 (10.7)	4 (3.6)	0 (0.0)	9 (22.0)	30 (10.5)	11 (4.5)
*Missing*	12 (2.1)	7 (6.1)	3 (2.5)	1 (0.9)	0 (0.0)	1 (0.9)	0 (0.0)	6 (2.1)	6 (2.5)
**ECOG performance score**,** n (%)**									
*0*	159 (27.7)	9 (7.8)	18 (14.8)	31 (27.7)	53 (48.2)	48 (41.7)	12 (29.3)	90 (31.4)	57 (23.4)
*1*	240 (41.8)	45 (39.1)	51 (41.8)	53 (47.3)	43 (39.1)	48 (41.7)	19 (46.3)	112 (39.0)	109 (44.7)
*2*	91 (15.9)	28 (24.3)	33 (27.0)	12 (10.7)	8 (7.3)	10 (8.7)	7 (17.1)	34 (11.8)	50 (20.5)
*Missing*	84 (14.6)	33 (28.7)	20 (16.4)	16 (14.3)	6 (5.5)	9 (7.8)	3 (7.3)	51 (17.8)	28 (11.5)
**Treatment category**,** n (%)**									
*Pre-treatment*	113 (19.7)	19 (16.5)	9 (7.4)	34 (30.4)	29 (26.4)	22 (19.1)	11 (26.8)	70 (24.4)	32 (13.1)
*On active treatment*	401 (69.9)	84 (73.0)	101 (82.8)	69 (61.6)	77 (70.0)	70 (60.9)	25 (61.0)	181 (63.1)	194 (79.5)
*Post-treatment survivorship*	60 (10.5)	12 (10.4)	12 (9.8)	9 (8.0)	4 (3.6)	23 (20.0)	5 (12.2)	36 (12.5)	18 (7.4)
**Treatment undertaken**,** n (%)**									
*No treatment*	113 (19.7)	19 (16.5)	9 (7.4)	34 (30.4)	29 (26.4)	22 (19.1)	11 (26.8)	70 (24.4)	32 (13.1)
*Surgery*	83 (14.5)	15 (13.0)	24 (19.7)	14 (12.5)	14 (12.7)	16 (13.9)	7 (17.1)	52 (18.1)	23 (9.4)
*Chemotherapy*	69 (12.0)	17 (14.8)	15 (12.3)	20 (17.9)	5 (4.5)	12 (10.4)	4 (9.8)	27 (9.4)	38 (15.6)
*Radiotherapy*	27 (4.7)	8 (7.0)	6 (4.9)	4 (3.6)	6 (5.5)	3 (2.6)	0 (0.0)	7 (2.4)	20 (8.2)
*Radiotherapy*,* Chemotherapy*	122 (21.3)	17 (14.8)	28 (23.0)	20 (17.9)	32 (29.1)	25 (21.7)	13 (31.7)	61 (21.3)	48 (19.7)
*Surgery*,* Radiotherapy*	39 (6.8)	10 (8.7)	12 (9.8)	4 (3.6)	7 (6.4)	6 (5.2)	1 (2.4)	19 (6.6)	19 (7.8)
*Surgery*,* Chemotherapy*	33 (5.7)	6 (5.2)	10 (8.2)	4 (3.6)	4 (3.6)	9 (7.8)	1 (2.4)	15 (5.2)	16 (6.6)
*Surgery*,* Radiotherapy*,* Chemotherapy*	83 (14.5)	21 (18.3)	16 (13.1)	12 (10.7)	13 (11.8)	21 (18.3)	3 (7.3)	33 (11.5)	47 (19.3)
*Missing*	5 (0.9)	2 (1.7)	2 (1.6)	0 (0.0)	0 (0.0)	1 (0.9)	1 (2.4)	3 (1.0)	1 (0.4)
**Comorbidities**,** n (%)**	178 (31.0)	26 (22.6)	33 (27.0)	26 (23.2)	53 (48.2)	40 (34.8)	11 (26.8)	81 (28.2)	86 (35.2)
**QOL score**,** Mean (SD)**	58 (15)	38	50	57	64	80	65	59	55
**Financial toxicity score**,** Median (IQR)**	16 (9–21)	10	19	19	14	17	28	20	8

Data represents distribution prior to imputation.

The most common gynecological malignancies were cervical cancer (*n* = 314, 54.7%) and ovarian cancer (*n* = 114, 19.9%), and most women had either stage 2 (*n* = 192, 33.4%) or stage 3 (*n* = 144, 25.1%) disease with ECOG performance scores of 1 (*n* = 240, 41.8%) or 0 (*n* = 159, 27.7%). Most women presented with a first tumor (*n* = 457, 79.6%) and were undergoing treatment at the time of the survey (*n* = 401, 69.9%). Of those undergoing treatment, the prevailing subcategories were radiotherapy and chemotherapy (*n* = 122, 21.3%), surgery alone (*n* = 83, 14.5%), or a combination of surgery, radiotherapy, and chemotherapy (*n* = 83, 14.5%). The prevalence of at least one comorbid condition among the enrolled patients was 31.0% (*n* = 178). Of these, the most common comorbidity was hypertension (*n* = 128, 71.9%) (**Table S3**). The stage of disease, treatment category, and presence of comorbidities all varied significantly by cancer type (**Table S2**).

### Health-related quality of life

The overall mean (SD) HRQoL score was 58 (15), and this tended to vary by cancer type (**Table S2**), with cervical cancer patients having a higher mean HRQoL score (60 ± 15) than other cancers and with the lowest mean HRQoL score among vulvar cancer patients (53 ± 12). Patients had the highest mean HRQoL subscale score in the social/family well-being domain (16.3 ± 5.5) and the lowest mean subscale score in the functional well-being domain (12.6 ± 5.4). Apart from the social/family well-being domain, all other subscales varied by cancer type (**Table S4**).

The results of the multivariable regression analysis on HRQoL scores are presented in Table 2. After multivariable adjustments, unemployed patients had lower mean HRQoL scores than employed patients (β = -2.4; 95%CI: -4.8, -0.02; *p* = 0.048). Compared to patients with cervical cancer, those with ovarian (β = -3.4; 95%CI: -6.4, -0.37; *p* = 0.028) and uterine cancers (β = -3.8; 95%CI: -7.0, -0.57; *p* = 0.021) and choriocarcinoma (β = -7.8; 95%CI: -15 -0.18; *p* = 0.045) had significantly lower mean HRQoL scores. In addition, compared to patients with stage I disease, the mean HRQoL score was significantly lower in patients with stage II (β = -4.6; 95%CI: -7.7, -1.6; *p* = 0.003), stage III (β = -5.5, 95%CI: -8.9, -2.2; *p* = 0.001), and stage IV disease (β = -4.6; 95%CI: -8.7, -0.41; *p* = 0.031). Furthermore, compared to an ECOG performance score of zero, the mean HRQoL score was significantly lower in patients with ECOG scores of 1 (β = -5.2; 95%CI: -7.9, -2.5; *p* < 0.001) and 2 (β = -12; 95%CI: -15, -8.0; *p* < 0.001). Finally, compared to those in the pre-treatment category, patients in post-treatment had significantly higher mean HRQoL scores (β = 9.3; 95CI: 5.0, 14; *p* < 0.001).

**Table 2:** Multivariable linear regression of health-related quality of life overall by sociodemographic and clinical characteristics (*N* = 572).

Characteristic	Unadjusted		Adjusted	
	Βeta (95% CI^1^)	*p*-value	Βeta (95% CI)	*p*-value
**Intercept**	-	-	70 (64, 77)	< 0.001
**Age**	-0.03 (-0.12, 0.06)	0.5	-0.03 (-0.13, 0.07)	0.6
**Centre**				
*LUTH*	Ref		Ref	
*LASUTH*	-11 (-14, -8.0)	< 0.001	-11 (-14, -7.5)	< 0.001
*UNTH*	1.4 (-1.8, 4.6)	0.4	0.76 (-3.0, 4.5)	0.7
*UBTH*	-0.78 (-5.0, 3.5)	0.7	2.3 (-2.8, 7.4)	0.4
*UCTH*	-4.5 (-11, 1.8)	0.2	-1.7 (-8.1, 4.8)	0.6
**Religion**				
*Christianity*	Ref		Ref	
*Islam*	-3.2 (-6.4, 0.02)	0.052	1.9 (-1.4, 5.1)	0.3
*Other*	-12 (-22, -2.5)	0.014	-0.38 (-9.3, 8.5)	> 0.9
**Ethnicity**				
*Yoruba*	Ref		Ref	
*Igbo*	4.9 (2.1, 7.8)	< 0.001	2.5 (-0.56, 5.6)	0.11
*Hausa*	-3.7 (-9.8, 2.4)	0.2	-4.4 (-10, 1.3)	0.13
*Other*	3.8 (0.62, 7.0)	0.019	1.7 (-1.8, 5.2)	0.3
**Marital Status**				
*Married*	Ref		Ref	
*Single*	-0.99 (-5.4, 3.4)	0.7	-0.15 (-4.4, 4.1)	> 0.9
*Widowed/Separated/Divorced*	-5.6 (-8.5, -2.7)	< 0.001	-2.5 (-5.2, 0.32)	0.083
**Level of Education**				
*Tertiary*	Ref		-	-
*Secondary*	0.34 (-2.4, 3.1)	0.8	-	-
*Primary*	-0.93 (-4.4, 2.6)	0.6	-	-
*None*	4.0 (-2.4, 10)	0.2	-	-
**Employment status**				
*Employed*	Ref		Ref	
*Unemployed*	-3.1 (-5.6, -0.61)	0.015	-2.4 (-4.8, -0.02)	0.048
**Income** ^*^	0.03 (-0.04, 0.01)	0.2	-	-
**On health insurance**			-	-
*No*	Ref		-	-
*Yes*	2.4 (-1.7, 6.4)	0.2	-	-
**Diagnosis**				
*Cervical cancer*	Ref		Ref	
*Ovarian cancer*	-4.7 (-7.9, -1.5)	0.004	-3.4 (-6.4, -0.37)	0.028
*Uterine cancer*	-3.5 (-7.1, -0.04)	0.047	-3.8 (-7.0, -0.57)	0.021
*Vulvar cancer*	-6.6 (-11, -1.8)	0.007	-3.7 (-8.0, 0.57)	0.089
*Choriocarcinoma*	-4.4 (-12, 3.2)	0.3	-7.8 (-15, -0.18)	0.045
**Stage**				
*Stage 1*	Ref		Ref	
*Stage 2*	-4.6 (-7.9, -1.3)	0.006	-4.6 (-7.7, -1.6)	0.003
*Stage 3*	-5.7 (-9.2, -2.3)	0.001	-5.5 (-8.9, -2.2)	0.001
*Stage 4*	-7.0 (-11, -2.7)	0.001	-4.6 (-8.7, -0.41)	0.031
**ECOG performance score**				
*0*	Ref		Ref	
*1*	-7.2 (-9.9, -4.6)	< 0.001	-5.2 (-7.9, -2.5)	< 0.001
*2*	-13 (-16, -9.8)	< 0.001	-12 (-15, -8.0)	< 0.001
**Diagnosis category**				
*First tumor*	Ref		Ref	
*Relapse/Recurrence*	-9.2 (-13, -5.3)	< 0.001	-3.0 (-6.9, 0.87)	0.13
*Second tumor*	-9.5 (-14, -5.3)	< 0.001	-4.2 (-8.3, 0.00)	0.050
**Treatment category**	-		-	
*Pre-treatment*	Ref		Ref	
*On active treatment*	-2.0 (-5.1, 1.1)	0.2	1.4 (-1.5, 4.3)	0.3
*Post-treatment*	4.7 (-0.01, 9.3)	0.051	9.3 (5.0, 14)	< 0.001
**Comorbidities**				
*No*	Ref		Ref	
*Yes*	3.2 (0.54, 5.8)	0.018	1.8 (-0.69, 4.2)	0.2

^1^CI = Confidence Interval, ^*^β per ₦1000.

### Financial toxicity

The overall median FACIT-COST score was 16 (IQR: 9–21), and this also tended to vary by cancer type, with cervical cancer patients having the lowest median scores (14, IQR: 8–21) and choriocarcinoma patients having the highest median scores (22, IQR: 20–23) (**Table S4**). Most patients (*n* = 531, 92.8%) experienced financial toxicity (FT), with more than a third (42.6%, *n* = 244) experiencing moderate-severe FT.

The results of multivariable regression on FT are shown in Table 3 and **Table S5**. After multivariable adjustments, older age was associated with better FT (β per 10-year increase in age = 0.6; 95%CI: 0.1, 1.1; *p* = 0.029), where a higher FACIT-COST score corresponds to better FT. Higher daily household income was also significantly associated with better FT (β per 1000 Naira = 0.02; 95%CI: 0.01, 0.03; *p* = 0.004). Compared to patients without health insurance, those on a form of health insurance had significantly better FT (β = 3.4; 95%CI: 1.4, 5.5; *p* = 0.001). Also, compared to patients with cervical cancer, those with ovarian cancer (β = 1.9; 95%CI: 0.29, 3.6; *p* = 0.021) and choriocarcinoma (β = 6.7; 95%CI: 2.6, 11; *p* = 0.001) had significantly better FT. In addition, compared to patients with stage I disease, FT was significantly worse in patients with stage III (β = -2.2; 95%CI: -4.0, -0.42; *p* = 0.016) and IV disease (β = -5.4; 95%CI: -7.6, -3.2; *p <* 0.001). Furthermore, compared to those in the pre-treatment category, those on active treatment had significantly worse FT (β = -2.9; 95%CI: -4.4, -1.3; *p <* 0.001). Finally, upon separate adjustment for treatment type, those who had radiotherapy alone (β = -6.7; 95%CI: -9.7, -3.7; *p* < 0.001) and a combination of surgery, radiotherapy and chemotherapy (β = -4.2; 95%CI: -6.3, -2.2; *p* < 0.001) had significantly worse FT compared to those who had no treatment (**Table S5**).

**Table 3:** Multivariable linear regression of financial toxicity by sociodemographic and clinical characteristics (*N* = 572).

Characteristic	Unadjusted		Adjusted	
	Βeta (95% CI^1^)	*p*-value	Βeta (95% CI)	*p*-value
**Intercept**				
**Age**	-0.02 (-0.07, 0.03)	0.4	0.06 (0.01, 0.11)	0.029
**Centre**				
*LUTH*	Ref		Ref	
*LASUTH*	-3.6 (-5.1, -2.0)	< 0.001	-4.9 (-6.6, -3.2)	< 0.001
*UNTH*	-8.1 (-9.8, -6.5)	< 0.001	-7.5 (-9.4, -5.6)	< 0.001
*UBTH*	-5.0 (-7.2, -2.8)	< 0.001	-6.0 (-8.5, -3.4)	< 0.001
*UCTH*	-5.0 (-8.4, -1.7)	0.003	-4.5 (-7.9, -1.0)	0.011
**Religion**				
*Christianity*	Ref		Ref	
*Islam*	2.5 (0.74, 4.2)	0.005	0.78 (-1.0, 2.6)	0.4
*Other*	-2.7 (-8.0, 2.6)	0.3	-0.26 (-5.1, 4.6)	> 0.9
**Ethnicity**				
*Yoruba*	Ref		Ref	
*Igbo*	-3.1 (-4.6, -1.6)	< 0.001	0.61 (-1.1, 2.3)	0.5
*Hausa*	-1.5 (-4.8, 1.8)	0.4	-0.12 (-3.2, 2.9)	> 0.9
*Other*	-2.3 (-4.1, -0.62)	0.008	-0.08 (-2.0, 1.9)	> 0.9
**Marital Status**				
*Married*	Ref		-	-
*Single*	-0.73 (-3.1, 1.7)	0.6	-	-
*Widowed/Separated/Divorced*	-1.0 (-2.6, 0.57)	0.2	-	-
**Level of Education**				
*Tertiary*	Ref		Ref	
*Secondary*	-1.7 (-3.2, -0.19)	0.027	-1.2 (-2.6, 0.15)	0.081
*Primary*	-2.9 (-4.7, -1.0)	0.003	-1.4 (-3.2, 0.42)	0.13
*None*	-2.3 (-5.7, 1.2)	0.2	-0.85 (-4.0, 2.3)	0.6
**Employment status**				
*Employed*	Ref		-	-
*Unemployed*	-0.71 (-2.0, 0.62)	0.3	-	-
**Income** ^*^	0.03 (0.01, 0.04)	< 0.001	0.02 (0.01, 0.03)	0.004
**On health insurance**				
*No*	Ref		Ref	
*Yes*	3.4 (1.2, 5.5)	0.002	3.4 (1.4, 5.5)	0.001
**Diagnosis**				
*Cervical cancer*	Ref		Ref	
*Ovarian cancer*	1.4 (-0.30, 3.1)	0.11	1.9 (0.29, 3.6)	0.021
*Uterine cancer*	3.3 (1.4, 5.1)	< 0.001	1.5 (-0.23, 3.2)	0.089
*Vulvar cancer*	1.0 (-1.5, 3.6)	0.4	1.6 (-0.75, 3.9)	0.2
*Choriocarcinoma*	6.2 (2.1, 10)	0.003	6.7 (2.6, 11)	0.001
**Stage**				
*Stage 1*	Ref		Ref	
*Stage 2*	-1.2 (-2.9, 0.60)	0.2	-1.6 (-3.3, 0.02)	0.053
*Stage 3*	-2.1 (-4.0, -0.27)	0.025	-2.2 (-4.0, -0.42)	0.016
*Stage 4*	-5.3 (-7.5, -3.0)	< 0.001	-5.4 (-7.6, -3.2)	< 0.001
**ECOG performance score**				
*0*	Ref		-	-
*1*	-1.3 (-2.8, 0.23)	0.10	-	-
*2*	-2.2 (-4.1, -0.38)	0.019^†^	-	-
**Diagnosis category**				
*First tumor*	Ref		Ref	
*Relapse/Recurrence*	0.91 (-1.3, 3.1)	0.4	1.3 (-0.79, 3.4)	0.2
*Second tumor*	3.8 (1.5, 6.1)	0.001	1.7 (-0.58, 4.0)	0.14
**Treatment category**				
*Pre-treatment*	Ref		Ref	
*On active treatment*	-3.5 (-5.1, -1.8)	< 0.001	-2.9 (-4.4, -1.3)	< 0.001
*Post-treatment*	-0.32 (-2.8, 2.2)	0.8	0.46 (-1.9, 2.8)	0.7
**Comorbidities**				
*No*	Ref		-	-
*Yes*	-1.2 (-2.7, 0.18)	0.087	-	-

^1^CI = Confidence Interval, ^*^β per ₦1000, ^†^Global *p*-value = 0.052.

### Effect of financial toxicity on Health-related quality of life

**Fig. 2:**
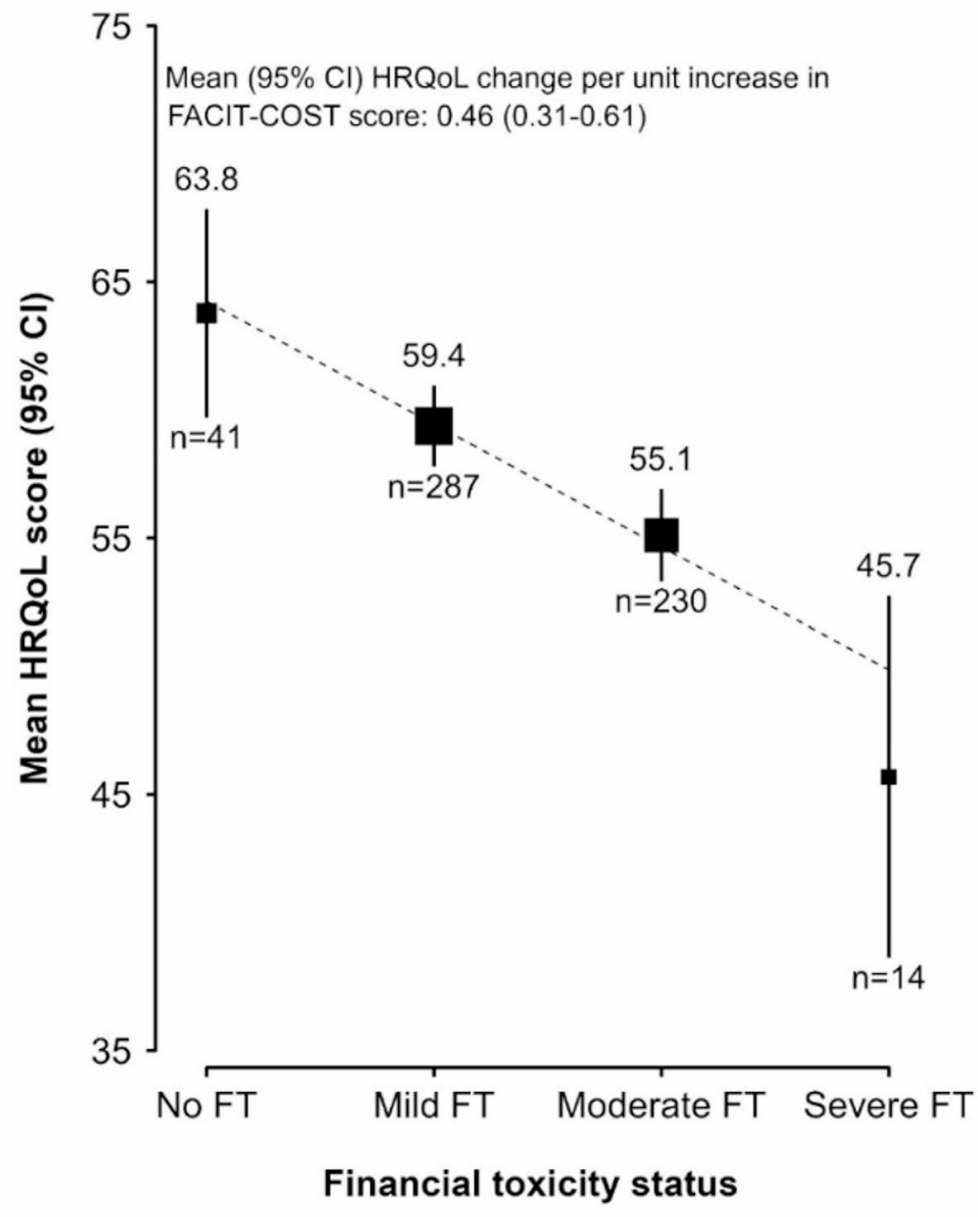
Effect of financial toxicity on health-related quality of life of patients. Association of health-related quality of life (HRQoL) with pre-defined groups of financial toxicity score, adjusted for age, center, religion, ethnicity, diagnosis, stage, diagnosis category (first tumor, relapse, recurrence, second tumor), and treatment category (pre-treatment, on active treatment, post-treatment survivorship). Marginal means of HRQoL scores are plotted against the medians of each financial toxicity group. The squares are weighted by the inverse of the group-specific variance. The numbers above and below each square represent the marginal means and the number of participants, respectively. Error bars are 95% confidence intervals. The dashed line corresponds to the slope of a weighted linear regression of group-specific medians on marginal means of HRQoL, where the weights are derived from the inverse of the group-specific variance.

**Fig. 3:**
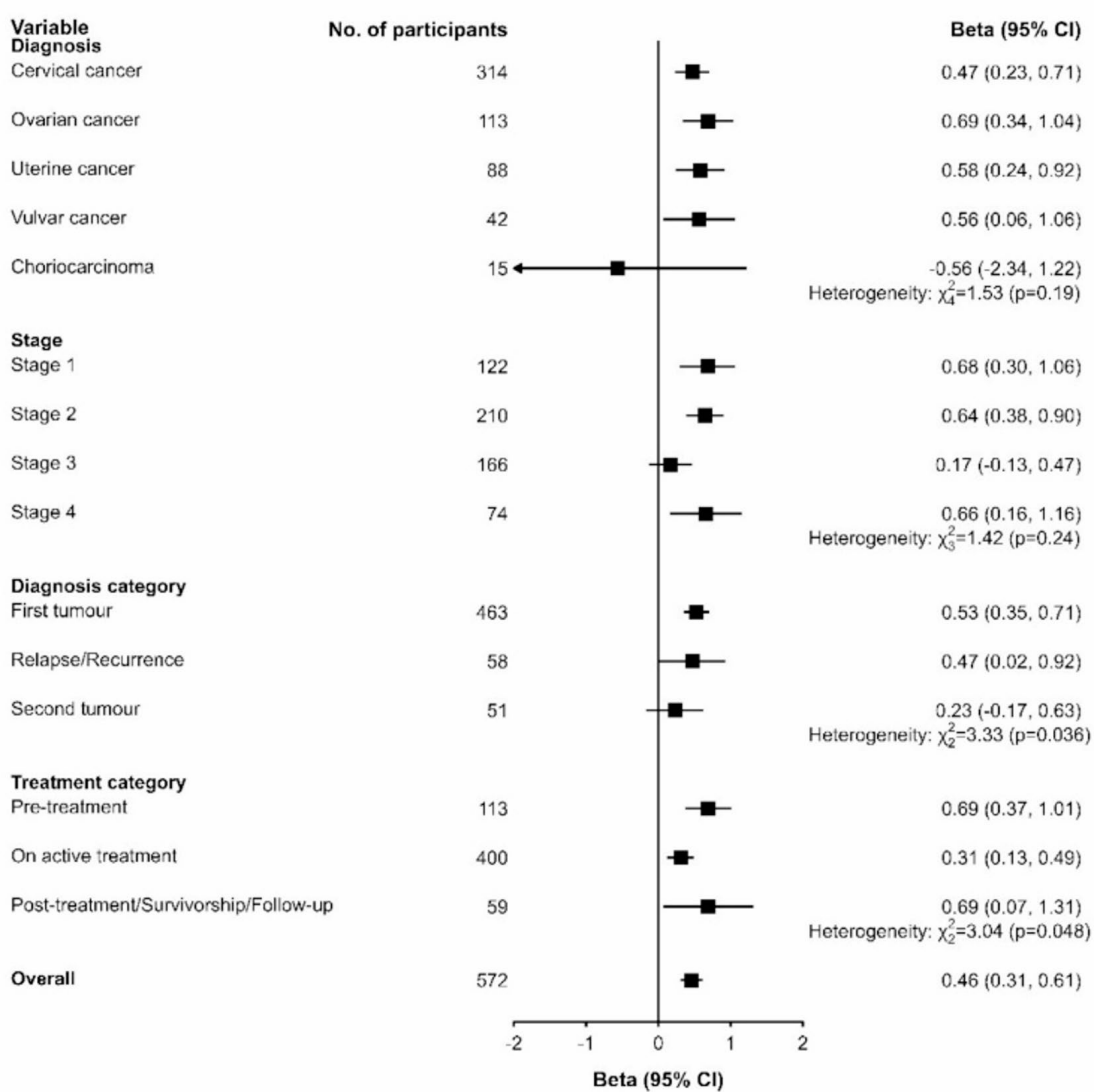
Multivariable-adjusted effect of financial toxicity on health-related quality of life by diagnosis, stage, diagnosis category and treatment category of respondents.

After multivariable adjustments, there was a linear relationship between FT groups and HRQoL scores (*p* = 0.426 for nonlinearity), indicating that better FT was associated with better HRQoL (Fig. 2). Each unit increase in FACIT-COST score (better FT) was significantly associated with a 0.46-unit higher mean HRQoL score (95%CI: 0.31, 0.61; *p* < 0.001). The effect of FT on HRQoL did not differ significantly by diagnosis (*p*_het_=0.192), or disease stage (*p*_het_=0.237) but differed significantly by diagnosis category (*p*_het_=0.036) and treatment category (*p*_het_=0.048) (Fig. 3). The effect of FT score on HRQoL was higher in those with a first tumor (β = 0.53; 95% CI: 0.35, 0.71; *p <* 0.001), those in pre-treatment (β = 0.69, 95% CI: 0.37, 1.02; *p <* 0.001) and those in post-treatment phases of care (β = 0.69, 95% CI: 0.04, 1.33; *p =* 0.037). Sensitivity analyses using multiple imputation did not materially alter the regression estimates (**Table S6-S9)**.

## Discussion

In the largest relevant study to date in Nigeria, we assessed the HRQoL and experience of FT among 574 Nigerian women with one of five gynecological malignancies across five tertiary facilities in southern Nigeria. We identified multivariable-adjusted associations between patient sociodemographic and clinical characteristics with both HRQoL and FT, including diagnosis, disease stage, and treatment category. Factors additionally associated with HRQoL included employment status, ECOG performance status, and diagnosis category. Furthermore, FT was associated with age, health insurance status, income, and treatment type. Also, we estimated a linear association between better FT and better overall HRQoL after adjusting for potential confounders, with the effect of FT on HRQoL significantly stronger for women with a first tumor and for those before and after treatment.

Our finding of worse HRQoL among unemployed women aligns with previous studies showing better HRQoL in employed cancer survivors^39^. Consistent with existing evidence, HRQoL also varied by cancer type in this study, with worse HRQoL in women with ovarian cancer – gynecological cancer with the poorest survival outcomes - as well as in those with uterine cancer and choriocarcinoma, compared to cervical cancer^4^. As expected, patients with higher disease stages and worse ECOG performance status had lower HRQoL, likely due to a more significant symptom burden, intensive treatments and poorer prognosis^40^. Although HRQoL did not differ significantly between women in pre-treatment and those on active treatment, it improved significantly in those who were in remission, likely underscoring the positive effects of successful treatment. Previous studies have similarly reported a better quality of life in cancer survivors up to 12 months post-treatment compared to baseline^41,42^.

Additionally, we identified previously established risk factors for worse FT, including younger age, lower education, lack of health insurance, lower income and cervical cancer diagnosis^9,15,31,43,44^. Younger patients may be more vulnerable to FT due to fewer accumulated earnings and the financial distress caused by decreased productivity^43^. Similarly, a cervical cancer diagnosis may reflect limited access to healthcare and preventive screening services, which in turn are markers of lower socioeconomic status, a known risk factor for greater financial toxicity^31^. Although no disease stages have been consistently associated with FT, our study found that a higher disease stage was associated with worse FT^9^. Furthermore, consistent with previous studies, our analyses show that patients on active treatment had worse FT than those in pre-treatment, with differences observed in FT by treatment type^29^. Access to cancer treatment remains a significant challenge for patients in Nigeria, where the majority must pay out-of-pocket for expensive therapies. Many essential treatments, such as radiotherapy, are not readily available, forcing patients to incur additional costs, including travel expenses for frequent medical visits^29,45,46^. Notably, most respondents in this study experienced FT, with more than a third of participants reporting moderate-severe FT, which is much higher than has been reported in other populations^31,43,44^.

Finally, we demonstrate a linear association between FT and the HRQoL of gynecological cancer patients, which persists even after adjusting for potential confounders, as reported in other populations^16,19,29^. In essence, better FT was associated with better HRQoL. This effect did not differ significantly by cancer type or disease stage in this study but was weaker in patients on active treatment and stronger in patients before and after treatment. This pattern could be explained by the fact that other factors, such as the adverse effects of treatments, may have a more significant impact on HRQoL in patients undergoing active treatment. In contrast, the HRQoL of those in the pre-treatment phase may be much more impacted by the acute distress resulting from the anticipated financial costs of care. Regardless, these findings highlight that financial toxicity persists throughout the care continuum and extends into the post-treatment phase, as patients may face ongoing financial challenges after exhausting available funds on treatment^16,29^.

This study is unique in that it is the largest in Nigeria to investigate HRQoL and FT among gynecological cancer patients, allowing us to identify meaningful differences within this population. We enhance the generalizability of our findings by recruiting patients from five tertiary centers and further strengthen our analysis by providing multivariable-adjusted estimates to reduce the effect of confounders. Additionally, we demonstrate the impact of FT on HRQoL, both overall and across subpopulations along the care continuum.

However, this study has certain limitations. First, the convenience sampling methodology and our inability to estimate enrolment rates due to logistical constraints make our findings susceptible to selection bias, as it is unclear whether our sample is representative of the underlying population. Second, self-reported patient characteristics potentially subject our study to information bias. Third, the cross-sectional design cannot guarantee the temporal relationship between identified predictors and the outcomes, making our associations susceptible to reverse causality. Fourth, while our multicenter recruitment improves generalizability, our findings may not be representative of the entire country. In particular, as the study included only participants who were proficient in English, the results may not fully capture the experiences of non-English-speaking individuals, who may differ in educational level or socioeconomic status. Fifth, although we adjusted for several patient and disease characteristics, the observational nature of this study cannot rule out unmeasured and residual confounding. In particular, some variables such as employment status were collapsed into broader categories to preserve statistical power, which may have masked heterogeneity within subgroups and limited our ability to fully adjust for their potential confounding effects. Finally, while the FACIT-COST tool is a validated measure of financial toxicity, it was initially developed for patients receiving ongoing treatment. Its applicability to patients in pre-treatment or post-treatment survivorship may be limited; therefore, findings related to these subgroups should be interpreted with caution.

Nevertheless, our study underscores the need for future more representative, nationwide studies of HRQoL and FT among gynecological cancer patients. Integrating these patient-reported outcomes into clinical assessment and management could enhance the quality of care for this population.

In conclusion, our findings identify sociodemographic and clinical factors associated with HRQoL and FT. Notably, we demonstrate a linear relationship between FT and HRQoL. These findings highlight the impact of these factors on patient management in low-resource settings and underscore their relevance in policy-making. Furthermore, our findings underscore the importance of integrating HRQoL and FT assessments into patient care, providing a foundation for future research in this area.

## Supplementary Information

Below is the link to the electronic supplementary material.

Supplementary Material 1

## Data Availability

The data underlying this article will be shared upon request to the corresponding author.

## Abbreviations

HRQoL: Health-related Quality of Life
FT: Financial Toxicity
LUTH: Lagos University Teaching Hospital
NSIA: Nigeria Sovereign Investment Authority
LASUTH: Lagos State University Teaching Hospital
UNTH: University of Nigeria Teaching Hospital
UCTH: University of Calabar Teaching Hospital
UBTH: University of Benin Teaching Hospital
FACT-G: Functional Assessment of Cancer Therapy - General
FACIT-COST: Functional Assessment of Chronic Illness Therapy Comprehensive Score for Financial Toxicity
ECOG: Eastern Co-operative Oncology Group
