# Supplementary material for: Factors associated with health-related quality of life and financial toxicity among gynecological cancer patients in Southern Nigeria
Source: Sci Rep. 2025 Jul 31;15:28041. doi: 10.1038/s41598-025-13763-0 (PMC12313957; doi:10.1038/s41598-025-13763-0)
Supplement: Supplementary file 1 — Supplementary Material 1 [file 41598_2025_13763_MOESM1_ESM.docx]

Supplementary methods and results

**Table of Contents**

[Appendix S1. Description of imputation procedure 2](#_Toc195176623)

[Table S1. STROBE Statement—Checklist of items that should be included in reports of *cross-sectional studies* 3](#_Toc195176624)

[Table S2. Baseline characteristics of participants by cancer type 5](#_Toc195176625)

[Table S3. Frequency of comorbid conditions reported 9](#_Toc195176626)

[Table S4*.* Health-related quality of life subscales by cancer type 10](#_Toc195176627)

[Table S5*.* Multivariable linear regression of financial toxicity additionally adjusted for treatment type 11](#_Toc195176628)

[Table S6. Results of multiple imputation for Multivariable linear regression of health-related quality of Life overall by sociodemographic and clinical characteristics 13](#_Toc195176629)

[Table S7. Results of multiple imputation for Multivariable linear regression of financial toxicity by sociodemographic and clinical characteristics 15](#_Toc195176630)

[Table S8. Results of multiple imputation for Multivariable linear regression of financial toxicity by sociodemographic and clinical characteristics additionally adjusted for treatment type 17](#_Toc195176631)

[Table S9. Results of single regression and multiple imputation for the effect of FT groups on HRQoL score after adjustments 19](#_Toc195176632)

[References 20](#_Toc195176633)

## Appendix S1. Description of imputation procedure

As a main analysis, single regression imputations were done for age, income, ECOG performance score, stage of disease, disease category, and treatment type. Imputations were done with one imputed dataset using the multivariate imputation by chained equations package (mice) in R.[1] Values were imputed for age (continuous) and income (continuous) using predictive mean matching, ECOG performance score (ordinal), and stage (ordinal) using polytomous logistic regression for ordered data (polr), and for diagnosis category and treatment type using polytomous logistic regression for nominal data (polyreg). We investigated patterns of missingness using logistic regression with a binary missingness indicator variable as the outcome and adjusting for all other variables as predictors, which revealed that missing values were not completely at random (MCAR). Missingness in age was significantly associated with center, religion, ethnicity, marital status, employment status, insurance status, diagnosis, presence of comorbidities, and treatment type (all p<0.05). Missingness in income was associated with center, educational status, employment status, diagnosis category, and total COST score (all p<0.05). Missingness in stage was significantly associated with center, education and total HRQoL score (all p<0.05).

To perform imputation, we included all variables used in the main analyses and other complete covariates in the data set whose information was not already captured in included covariates in the predictor matrix (e.g., type of insurance and degree of coverage were not used because information was already captured in insurance status). These predictors included age, centre, religion, ethnicity, marital status, education, income, ECOG performance score, employment status, insurance status, diagnosis, stage, diagnosis category, treatment category, treatment type, presence of comorbidities, total COST and HRQoL scores as well as item 12 in the COST tool which is not included in the total COST calculations.

As a sensitivity analysis, we performed multiple imputation using the mice package, using five imputed data sets with the same predictors as above and pooling the results according to Rubin’s method.[1,2]

## Table S1. STROBE Statement—Checklist of items that should be included in reports of *cross-sectional studies*

|  | Item No | Recommendation | Page No |
| --- | --- | --- | --- |
| **Title and abstract** | 1 | (*a*) Indicate the study’s design with a commonly used term in the title or the abstract | 1 |
|  |  | (*b*) Provide in the abstract an informative and balanced summary of what was done and what was found | 3-4 |
| Introduction | | | |
| Background/rationale | 2 | Explain the scientific background and rationale for the investigation being reported | 5-6 |
| Objectives | 3 | State specific objectives, including any prespecified hypotheses | 6 |
| Methods | | | |
| Study design | 4 | Present key elements of study design early in the paper | 7 |
| Setting | 5 | Describe the setting, locations, and relevant dates, including periods of recruitment, exposure, follow-up, and data collection | 7 |
| Participants | 6 | (*a*) Give the eligibility criteria, and the sources and methods of selection of participants | 7 |
| Variables | 7 | Clearly define all outcomes, exposures, predictors, potential confounders, and effect modifiers. Give diagnostic criteria, if applicable | 8-9 |
| Data sources/ measurement | 8* | For each variable of interest, give sources of data and details of methods of assessment (measurement). Describe comparability of assessment methods if there is more than one group | 8-9 |
| Bias | 9 | Describe any efforts to address potential sources of bias | 8-12 |
| Study size | 10 | Explain how the study size was arrived at | 8 |
| Quantitative variables | 11 | Explain how quantitative variables were handled in the analyses. If applicable, describe which groupings were chosen and why | 10-12 |
| Statistical methods | 12 | (*a*) Describe all statistical methods, including those used to control for confounding | 10-12 |
|  |  | (*b*) Describe any methods used to examine subgroups and interactions | 11-12 |
|  |  | (*c*) Explain how missing data were addressed | 10-12 |
|  |  | (*d*) If applicable, describe analytical methods taking account of sampling strategy | 10-12 |
|  |  | (*e*) Describe any sensitivity analyses | 11-12 |
| Results | | | |
| Participants | 13* | (a) Report numbers of individuals at each stage of study—eg numbers potentially eligible, examined for eligibility, confirmed eligible, included in the study, completing follow-up, and analyzed | 10-13 |
|  |  | (b) Give reasons for non-participation at each stage |  |
|  |  | (c) Consider use of a flow diagram |  |
| Descriptive data | 14* | (a) Give characteristics of study participants (eg demographic, clinical, social) and information on exposures and potential confounders | 13 |
|  |  | (b) Indicate number of participants with missing data for each variable of interest | 10 |
| Outcome data | 15* | Report numbers of outcome events or summary measures | 13 |
| Main results | 16 | (*a*) Give unadjusted estimates and, if applicable, confounder-adjusted estimates and their precision (eg, 95% confidence interval). Make clear which confounders were adjusted for and why they were included | 13-16, 33-34 |
|  |  | (*b*) Report category boundaries when continuous variables were categorized | 8-10 |
|  |  | (*c*) If relevant, consider translating estimates of relative risk into absolute risk for a meaningful time period | NA |
| Other analyses | 17 | Report other analyses done—eg analyses of subgroups and interactions, and sensitivity analyses | 11-12, |
| Discussion | | | |
| Key results | 18 | Summarise key results with reference to study objectives | 17-19 |
| Limitations | 19 | Discuss limitations of the study, taking into account sources of potential bias or imprecision. Discuss both direction and magnitude of any potential bias | 19 |
| Interpretation | 20 | Give a cautious overall interpretation of results considering objectives, limitations, multiplicity of analyses, results from similar studies, and other relevant evidence | 17-19 |
| Generalisability | 21 | Discuss the generalisability (external validity) of the study results | 18-19 |
| Other information | | | |
| Funding | 22 | Give the source of funding and the role of the funders for the present study and, if applicable, for the original study on which the present article is based | 23 |

*Give information separately for exposed and unexposed groups.

## Table S2. Baseline characteristics of participants by cancer type

| Characteristic | Cervical cancer  N = 314 | Ovarian cancer  N = 114 | Uterine cancer  N = 89 | Vulvar cancer  N = 42 | Choriocarcinoma  N = 15 |
| --- | --- | --- | --- | --- | --- |
| **Age (in years), Median (IQR)** | 56 (48 - 64) | 43 (34 - 51) | 55 (47 - 61) | 46 (34 - 62) | 25 (20 - 30) |
| **Centre, n (%)** |  |  |  |  |  |
| *LUTH* | 152 (48.4) | 39 (34.2) | 54 (60.7) | 17 (40.5) | 0 (0.0) |
| *LASUTH* | 53 (16.9) | 33 (28.9) | 18 (20.2) | 15 (35.7) | 13 (86.7) |
| *UNTH* | 75 (23.9) | 11 (9.6) | 12 (13.5) | 7 (16.7) | 0 (0.0) |
| *UBTH* | 23 (7.3) | 25 (21.9) | 4 (4.5) | 2 (4.8) | 0 (0.0) |
| *UCTH* | 11 (3.5) | 6 (5.3) | 1 (1.1) | 1 (2.4) | 2 (13.3) |
| **Religion, n (%)** |  |  |  |  |  |
| *Christianity* | 274 (87.3) | 89 (78.1) | 59 (66.3) | 32 (76.2) | 11 (73.3) |
| *Islam* | 37 (11.8) | 23 (20.2) | 27 (30.3) | 9 (21.4) | 4 (26.7) |
| *Other* | 3 (1.0) | 2 (1.8) | 3 (3.4) | 1 (2.4) | 0 (0.0) |
| **Ethnicity, n (%)** |  |  |  |  |  |
| *Yoruba* | 116 (36.9) | 51 (44.7) | 36 (40.4) | 13 (31.0) | 6 (40.0) |
| *Igbo* | 119 (37.9) | 29 (25.4) | 28 (31.5) | 13 (31.0) | 6 (40.0) |
| *Hausa* | 12 (3.8) | 2 (1.8) | 5 (5.6) | 5 (11.9) | 1 (6.7) |
| *Other/missing* | 67 (21.3) | 32 (28.1) | 20 (22.5) | 11 (26.2) | 2 (13.3) |
| **Marital status, n (%)** |  |  |  |  |  |
| *Married* | 215 (68.5) | 75 (65.8) | 66 (74.2) | 28 (66.7) | 8 (53.3) |
| *Single* | 15 (4.8) | 18 (15.8) | 3 (3.4) | 5 (11.9) | 7 (46.7) |
| *Widowed/Separated/Divorced* | 84 (26.8) | 21 (18.4) | 20 (22.5) | 9 (21.4) | 0 (0.0) |
| **Level of education, n (%)** |  |  |  |  |  |
| *None* | 15 (4.8) | 0 (0.0) | 7 (7.9) | 1 (2.4) | 0 (0.0) |
| *Primary* | 61 (19.4) | 16 (14.0) | 16 (18.0) | 5 (11.9) | 2 (13.3) |
| *Secondary* | 125 (39.8) | 33 (28.9) | 31 (34.8) | 16 (38.1) | 6 (40.0) |
| *Tertiary* | 113 (36.0) | 65 (57.0) | 35 (39.3) | 20 (47.6) | 7 (46.7) |
| **Daily household income (Naira), Median** | 3,000 | 3,333 | 3,550 | 3,000 | 20,000 |
| **Employment status, n (%)** |  |  |  |  |  |
| *Employed* | 170 (54.1) | 73 (64.0) | 56 (62.9) | 20 (47.6) | 13 (86.7) |
| *Unemployed* | 144 (45.9) | 41 (36.0) | 33 (37.1) | 22 (52.4) | 2 (13.3) |
| **On health insurance, n (%)** | 27 (8.6) | 14 (12.3) | 15 (16.9) | 3 (7.1) | 0 (0.0) |
| **Health insurance type, n (%)** |  |  |  |  |  |
| *Government e.g. NHIS* | 20 (74.1) | 7 (50.0) | 5 (33.3) | 1 (33.3) | 0 (NA) |
| *Private e.g company owned* | 3 (11.1) | 6 (42.9) | 10 (66.7) | 2 (66.7) | 0 (NA) |
| *Cancer Health Fund* | 1 (3.7) | 0 (0.0) | 0 (0.0) | 0 (0.0) | 0 (NA) |
| *Unknown* | 3 (11.1) | 1 (7.1) | 0 (0.0) | 0 (0.0) | 0 (NA) |
| **Insurance coverage of cancer treatment, n (%)** |  |  |  |  |  |
| *Not at all* | 4 (14.8) | 2 (14.3) | 0 (0.0) | 0 (0.0) | 0 (NA) |
| *Yes, Partly* | 19 (70.4) | 10 (71.4) | 13 (86.7) | 3 (100.0) | 0 (NA) |
| *Yes, Fully* | 1 (3.7) | 2 (14.3) | 1 (6.7) | 0 (0.0) | 0 (NA) |
| *Unknown* | 3 (11.1) | 0 (0.0) | 1 (6.7) | 0 (0.0) | 0 (NA) |
| **Stage, n (%)** |  |  |  |  |  |
| *Stage 1* | 49 (15.6) | 26 (22.8) | 15 (16.9) | 8 (19.0) | 11 (73.3) |
| *Stage 2* | 119 (37.9) | 28 (24.6) | 34 (38.2) | 10 (23.8) | 1 (6.7) |
| *Stage 3* | 89 (28.3) | 23 (20.2) | 20 (22.5) | 12 (28.6) | 0 (0.0) |
| *Stage 4* | 31 (9.9) | 19 (16.7) | 9 (10.1) | 5 (11.9) | 0 (0.0) |
| *Unknown* | 26 (8.3) | 18 (15.8) | 11 (12.4) | 7 (16.7) | 3 (20.0) |
| **Category of diagnosis, n (%)** |  |  |  |  |  |
| *First tumor* | 262 (83.4) | 86 (75.4) | 66 (74.2) | 30 (71.4) | 13 (86.7) |
| *Relapse/Recurrence* | 20 (6.4) | 14 (12.3) | 11 (12.4) | 8 (19.0) | 1 (6.7) |
| *Second tumor* | 26 (8.3) | 10 (8.8) | 11 (12.4) | 4 (9.5) | 0 (0.0) |
| *Unknown* | 6 (1.9) | 4 (3.5) | 1 (1.1) | 0 (0.0) | 1 (6.7) |
| **ECOG performance score, n (%)** |  |  |  |  |  |
| *0* | 96 (30.6) | 20 (17.5) | 29 (32.6) | 7 (16.7) | 7 (46.7) |
| *1* | 131 (41.7) | 46 (40.4) | 40 (44.9) | 22 (52.4) | 1 (6.7) |
| *2* | 45 (14.3) | 26 (22.8) | 13 (14.6) | 7 (16.7) | 0 (0.0) |
| *Unknown* | 42 (13.4) | 22 (19.3) | 7 (7.9) | 6 (14.3) | 7 (46.7) |
| **Treatment category, n (%)** |  |  |  |  |  |
| *Pre-treatment* | 63 (20.1) | 19 (16.7) | 21 (23.6) | 9 (21.4) | 1 (6.7) |
| *On active treatment* | 233 (74.2) | 80 (70.2) | 53 (59.6) | 29 (69.0) | 6 (40.0) |
| *Post-treatment/Survivorship/Follow-up* | 18 (5.7) | 15 (13.2) | 15 (16.9) | 4 (9.5) | 8 (53.3) |
| **Treatment undertaken, n (%)** |  |  |  |  |  |
| *No treatment* | 63 (20.1) | 19 (16.7) | 21 (23.6) | 9 (21.4) | 1 (6.7) |
| *Surgery* | 29 (9.2) | 29 (25.4) | 19 (21.3) | 5 (11.9) | 1 (6.7) |
| *Chemotherapy* | 23 (7.3) | 23 (20.2) | 6 (6.7) | 6 (14.3) | 11 (73.3) |
| *Radiotherapy* | 16 (5.1) | 3 (2.6) | 4 (4.5) | 4 (9.5) | 0 (0.0) |
| *Radiotherapy, Chemotherapy* | 97 (30.9) | 7 (6.1) | 13 (14.6) | 5 (11.9) | 0 (0.0) |
| *Surgery, Radiotherapy* | 18 (5.7) | 11 (9.6) | 5 (5.6) | 3 (7.1) | 2 (13.3) |
| *Surgery, Chemotherapy* | 13 (4.1) | 10 (8.8) | 7 (7.9) | 3 (7.1) | 0 (0.0) |
| *Surgery, Radiotherapy, Chemotherapy* | 53 (16.9) | 10 (8.8) | 13 (14.6) | 7 (16.7) | 0 (0.0) |
| *Unknown* | 2 (0.6) | 2 (1.8) | 1 (1.1) | 0 (0.0) | 0 (0.0) |
| **Comorbidities, n (%)** | 123 (39.2) | 26 (22.8) | 17 (19.1) | 11 (26.2) | 1 (6.7) |
| **QOL score, Mean (SD)** | 60 (15) | 55 (15) | 56 (15) | 53 (12) | 55 (9) |
| **Financial toxicity score, Median (IQR)** | 14 (8 - 21) | 17 (10 - 22) | 20 (11 - 23) | 16 (6 - 22) | 22 (20 - 23) |

## Table S3. Frequency of comorbid conditions reported

| **Comorbidity** | **n (%)** |
| --- | --- |
| Hypertension | 128 (71.9) |
| Diabetes mellitus | 35 (19.7%) |
| Peptic Ulcer Disease | 14 (7.9%) |
| HIV | 12 (6.7%) |
| Hepatitis B | 4 (2.2%) |
| Asthma | 3 (1.7%) |
| Kidney disease | 3 (1.7%) |
| Hepatitis C | 1 (0.6%) |
| Bipolar Affective Disorder | 1 (0.6%) |
| Dyslipidemia | 1 (0.6%) |
| Osteoarthritis | 1 (0.6%) |
| Thyroid disease | 1 (0.6%) |
| Back pain | 1 (0.6%) |

## Table S4*.* Health-related quality of life subscales by cancer type

| Characteristic [Mean (SD)] | Overall  N = 574 | Cervical  N = 314 | Ovarian  N = 114 | Uterine  N = 89 | Vulvar  N = 42 | Choriocarcinoma  N = 15 | p-value^1^ |
| --- | --- | --- | --- | --- | --- | --- | --- |
| **Physical well-being** | 15.1 (5.8) | 15.8 (5.8) | 14.5 (6.0) | 14.8 (5.3) | 13.6 (5.3) | 10.2 (4.2) | <0.001 |
| **Social/Family well-being** | 16.3 (5.5) | 16.6 (5.9) | 15.9 (5.5) | 16.2 (5.1) | 15.6 (4.6) | 16.5 (3.7) | 0.57 |
| **Emotional well-being** | 13.6 (4.8) | 14.2 (4.7) | 13.3 (4.7) | 12.7 (5.1) | 11.7 (4.2) | 14.6 (3.1) | 0.003 |
| **Functional well-being** | 12.6 (5.4) | 13.1 (5.4) | 11.2 (5.8) | 12.4 (5.3) | 12.2 (4.7) | 14.0 (3.0) | 0.021 |
| ^1^Kruskal-Wallis rank sum test | | | | | | | |

## Table S5*.* Multivariable linear regression of financial toxicity additionally adjusted for treatment type

| **Characteristic** | **Beta** | **95% CI**^1^ | **p-value** |
| --- | --- | --- | --- |
| **(Intercept)** | 17 | 14, 21 | <0.001 |
| **Age** | 0.06 | 0.01, 0.12 | 0.017 |
| **Centre** |  |  |  |
| *LUTH* | — | — |  |
| *LASUTH* | -4.7 | -6.5, -2.9 | <0.001 |
| *UNTH* | -7.2 | -9.1, -5.2 | <0.001 |
| *UBTH* | -5.8 | -8.3, -3.2 | <0.001 |
| *UCTH* | -5.0 | -8.5, -1.5 | 0.006 |
| **Religion** |  |  |  |
| *Christianity* | — | — |  |
| *Islam* | 0.77 | -1.0, 2.6 | 0.399 |
| *Other* | 1.0 | -3.8, 5.9 | 0.673 |
| **Ethnicity** |  |  |  |
| *Yoruba* | — | — |  |
| *Igbo* | 0.95 | -0.74, 2.6 | 0.271 |
| *Hausa* | 0.42 | -2.7, 3.5 | 0.791 |
| *Other* | 0.16 | -1.8, 2.1 | 0.870 |
| **Education** |  |  |  |
| *Tertiary* | — | — |  |
| *Secondary* | -1.3 | -2.7, 0.12 | 0.074 |
| *Primary* | -1.6 | -3.4, 0.22 | 0.085 |
| *None* | -0.84 | -4.0, 2.4 | 0.604 |
| **Income** | 0.02 | 0.01, 0.03 | 0.005 |
| **On health insurance** |  |  |  |
| *No* | — | — |  |
| *Yes* | 3.4 | 1.3, 5.4 | 0.001 |
| **Diagnosis** |  |  |  |
| *Cervical cancer* | — | — |  |
| *Ovarian cancer* | 1.8 | 0.07, 3.4 | 0.041 |
| *Uterine cancer* | 1.7 | -0.05, 3.4 | 0.057 |
| *Vulvar cancer* | 1.8 | -0.50, 4.2 | 0.124 |
| *Choriocarcinoma* | 7.9 | 3.7, 12 | <0.001 |
| **Stage** |  |  |  |
| *Stage 1* | — | — |  |
| *Stage 2* | -1.9 | -3.6, -0.24 | 0.025 |
| *Stage 3* | -2.2 | -4.0, -0.37 | 0.018 |
| *Stage 4* | -5.2 | -7.5, -3.0 | <0.001 |
| **Diagnosis category** |  |  |  |
| *First tumor* | — | — |  |
| *Relapse/Recurrence* | 1.5 | -0.58, 3.6 | 0.158 |
| *Second tumor* | 1.4 | -0.94, 3.8 | 0.240 |
| **Treatment type** |  |  |  |
| *No treatment* | — | — |  |
| *Surgery* | -0.65 | -2.7, 1.4 | 0.538 |
| *Chemotherapy* | -2.3 | -4.6, 0.01 | 0.051 |
| *Radiotherapy* | -6.7 | -9.7, -3.7 | <0.001 |
| *Surgery, Chemotherapy* | -2.4 | -5.2, 0.42 | 0.095 |
| *Radiotherapy, Chemotherapy* | -1.9 | -3.9, 0.07 | 0.059 |
| *Surgery, Radiotherapy* | -2.3 | -5.1, 0.46 | 0.102 |
| *Surgery, Radiotherapy, Chemotherapy* | -4.2 | -6.3, -2.2 | <0.001 |
| ^1^CI = Confidence Interval | | | |

## Table S6. Results of multiple imputation for Multivariable linear regression of health-related quality of Life overall by sociodemographic and clinical characteristics

| **Characteristic** | **Beta** | **95% CI**^1^ | **p-value** |
| --- | --- | --- | --- |
| **(Intercept)** | 68 | 61, 75 | <0.001 |
| **Age** | -0.01 | -0.13, 0.12 | >0.9 |
| **Centre** |  |  |  |
| *LUTH* | — | — |  |
| *LASUTH* | -11 | -14, -7.1 | <0.001 |
| *UNTH* | 0.50 | -3.2, 4.2 | 0.8 |
| *UBTH* | 2.1 | -3.1, 7.2 | 0.4 |
| *UCTH* | -1.3 | -7.9, 5.3 | 0.7 |
| **Religion** |  |  |  |
| *Christianity* | — | — |  |
| *Islam* | 1.9 | -1.4, 5.3 | 0.3 |
| *Other* | -0.54 | -9.7, 8.6 | >0.9 |
| **Ethnicity** |  |  |  |
| *Yoruba* | — | — |  |
| *Igbo* | 2.7 | -0.41, 5.9 | 0.088 |
| *Hausa* | -4.6 | -10, 1.1 | 0.11 |
| *Other* | 1.4 | -2.2, 5.0 | 0.4 |
| **Marital Status** |  |  |  |
| *Married* | — | — |  |
| *Single* | 0.33 | -4.0, 4.7 | 0.9 |
| *Widowed/Separated/Divorced* | -2.8 | -5.7, 0.07 | 0.055 |
| **Employment status** |  |  |  |
| *Employed* | — | — |  |
| *Unemployed* | -2.6 | -5.0, -0.22 | 0.032 |
| **Diagnosis** |  |  |  |
| *Cervical cancer* | — | — |  |
| *Ovarian cancer* | -3.2 | -6.3, -0.14 | 0.041 |
| *Uterine cancer* | -3.7 | -6.9, -0.42 | 0.027 |
| *Vulvar cancer* | -3.4 | -7.7, 0.97 | 0.13 |
| *Choriocarcinoma* | -7.2 | -15, 0.93 | 0.082 |
| **Stage** |  |  |  |
| *Stage 1* | — | — |  |
| *Stage 2* | -3.8 | -7.0, -0.60 | 0.020 |
| *Stage 3* | -4.9 | -8.4, -1.3 | 0.007 |
| *Stage 4* | -4.9 | -9.2, -0.56 | 0.027 |
| **ECOG score** |  |  |  |
| *0* | — | — |  |
| *1* | -5.0 | -7.9, -2.1 | <0.001 |
| *2* | -11 | -15, -7.1 | <0.001 |
| **Diagnosis category** |  |  |  |
| *First tumor* | — | — |  |
| *Relapse/Recurrence* | -2.4 | -6.4, 1.6 | 0.2 |
| *Second tumor* | -5.3 | -9.7, -0.78 | 0.022 |
| **Treatment category** |  |  |  |
| *Pre-treatment* | — | — |  |
| *On active treatment* | 1.4 | -1.5, 4.3 | 0.3 |
| *Post-treatment/Survivorship/Follow-up* | 9.2 | 4.8, 14 | <0.001 |
| **Comorbidities** |  |  |  |
| *No* | — | — |  |
| *Yes* | 1.8 | -0.73, 4.3 | 0.2 |
| ^1^CI = Confidence Interval | | | |

## Table S7. Results of multiple imputation for Multivariable linear regression of financial toxicity by sociodemographic and clinical characteristics

| **Characteristic** | **Beta** | **95% CI**^1^ | **p-value** |
| --- | --- | --- | --- |
| **(Intercept)** | 17 | 12, 21 | <0.001 |
| **Age** | 0.07 | 0.02, 0.12 | 0.005 |
| **Centre** |  |  |  |
| *LUTH* | — | — |  |
| *LASUTH* | -5.1 | -6.8, -3.4 | <0.001 |
| *UNTH* | -7.5 | -9.4, -5.6 | <0.001 |
| *UBTH* | -5.7 | -8.2, -3.1 | <0.001 |
| *UCTH* | -4.6 | -8.1, -1.1 | 0.011 |
| **Religion** |  |  |  |
| *Christianity* | — | — |  |
| *Islam* | 0.86 | -0.92, 2.6 | 0.3 |
| *Other* | -0.40 | -5.3, 4.5 | 0.9 |
| **Ethnicity** |  |  |  |
| *Yoruba* | — | — |  |
| *Igbo* | 0.73 | -0.96, 2.4 | 0.4 |
| *Hausa* | -0.22 | -3.3, 2.8 | 0.9 |
| *Other* | -0.08 | -2.0, 1.9 | >0.9 |
| **Education** |  |  |  |
| *None* | — | — |  |
| *Primary* | -0.68 | -3.9, 2.6 | 0.7 |
| *Secondary* | -0.44 | -3.5, 2.7 | 0.8 |
| *Tertiary* | 0.94 | -2.2, 4.1 | 0.6 |
| **Income^*^** | 0.02 | 0.0003  , 0.03 | 0.046 |
| **On health insurance** |  |  |  |
| *No* | — | — |  |
| *Yes* | 3.2 | 1.2, 5.2 | 0.002 |
| **Diagnosis** |  |  |  |
| *Cervical cancer* | — | — |  |
| *Choriocarcinoma* | 7.0 | 2.9, 11 | <0.001 |
| *Ovarian cancer* | 2.1 | 0.41, 3.7 | 0.014 |
| *Uterine cancer* | 1.4 | -0.29, 3.2 | 0.10 |
| *Vulvar cancer* | 1.5 | -0.82, 3.8 | 0.2 |
| *Choriocarcinoma* | 7.0 | 2.9, 11 | <0.001 |
| **Stage** |  |  |  |
| *Stage 1* | — | — |  |
| *Stage 2* | -1.8 | -3.7, 0.00 | 0.050 |
| *Stage 3* | -2.8 | -4.9, -0.74 | 0.009 |
| *Stage 4* | -5.7 | -7.9, -3.5 | <0.001 |
| **Diagnosis category** |  |  |  |
| *First tumor* | — | — |  |
| *Relapse/Recurrence* | 1.4 | -0.68, 3.6 | 0.2 |
| *Second tumor* | 1.9 | -0.44, 4.2 | 0.11 |
| **Treatment category** |  |  |  |
| *Pre-treatment* | — | — |  |
| *On active treatment* | -2.9 | -4.4, -1.3 | <0.001 |
| *Post-treatment/Survivorship/Follow-up* | 0.46 | -1.9, 2.8 | 0.7 |
| ^1^CI = Confidence Interval, ^*^β per ₦1000 | | | |

## Table S8. Results of multiple imputation for Multivariable linear regression of financial toxicity by sociodemographic and clinical characteristics additionally adjusted for treatment type

| **Characteristic** | **Beta** | **95% CI**^1^ | **p-value** |
| --- | --- | --- | --- |
| **(Intercept)** | 16 | 11, 21 | <0.001 |
| **Age** | 0.08 | 0.03, 0.13 | 0.002 |
| **Centre** |  |  |  |
| *LUTH* | — | — |  |
| *LASUTH* | -4.9 | -6.7, -3.0 | <0.001 |
| *UNTH* | -7.1 | -9.1, -5.2 | <0.001 |
| *UBTH* | -5.5 | -8.0, -2.9 | <0.001 |
| *UCTH* | -5.2 | -8.8, -1.6 | 0.005 |
| **Religion** |  |  |  |
| *Christianity* | — | — |  |
| *Islam* | 0.88 | -0.91, 2.7 | 0.3 |
| *Other* | 0.95 | -4.0, 5.8 | 0.7 |
| **Ethnicity** |  |  |  |
| *Yoruba* | — | — |  |
| *Igbo* | 1.1 | -0.63, 2.8 | 0.2 |
| *Hausa* | 0.25 | -2.8, 3.3 | 0.9 |
| *Other* | 0.18 | -1.8, 2.1 | 0.9 |
| **Education** |  |  |  |
| *None* | — | — |  |
| *Primary* | -0.88 | -4.1, 2.3 | 0.6 |
| *Secondary* | -0.47 | -3.6, 2.6 | 0.8 |
| *Tertiary* | 0.96 | -2.2, 4.1 | 0.6 |
| **Income^*^** | 0.02 | -0.0002  , 0.03 | 0.053 |
| **On health insurance** |  |  |  |
| *No* | — | — |  |
| *Yes* | 3.1 | 1.1, 5.2 | 0.003 |
| **Diagnosis** |  |  |  |
| *Cervical cancer* | — | — |  |
| *Choriocarcinoma* | 8.1 | 3.9, 12 | <0.001 |
| *Ovarian cancer* | 1.9 | 0.18, 3.5 | 0.030 |
| *Uterine cancer* | 1.6 | -0.17, 3.3 | 0.076 |
| *Vulvar cancer* | 1.7 | -0.58, 4.1 | 0.14 |
| **Stage** |  |  |  |
| *Stage 1* | — | — |  |
| *Stage 2* | -2.1 | -3.9, -0.30 | 0.022 |
| *Stage 3* | -2.9 | -4.9, -0.84 | 0.006 |
| *Stage 4* | -5.6 | -7.8, -3.4 | <0.001 |
| **Diagnosis category** |  |  |  |
| *First tumor* | — | — |  |
| *Relapse/Recurrence* | 1.7 | -0.38, 3.8 | 0.11 |
| *Second tumor* | 1.6 | -0.71, 4.0 | 0.2 |
| **Treatment type** |  |  |  |
| *No treatment* | — | — |  |
| *Surgery* | -0.63 | -2.7, 1.5 | 0.6 |
| *Chemotherapy* | -2.1 | -4.4, 0.24 | 0.079 |
| *Radiotherapy* | -6.6 | -9.7, -3.6 | <0.001 |
| *Surgery, Chemotherapy* | -2.5 | -5.4, 0.29 | 0.079 |
| *Radiotherapy, Chemotherapy* | -2.1 | -4.0, -0.06 | 0.043 |
| *Surgery, Radiotherapy* | -2.6 | -5.4, 0.22 | 0.071 |
| *Surgery, Radiotherapy, Chemotherapy* | -4.4 | -6.5, -2.3 | <0.001 |
| ^1^CI = Confidence Interval, ^*^β per ₦1000 | | | |

## Table S9. Results of single regression and multiple imputation for the effect of FT groups on HRQoL score after adjustments

| **Variable** | **Number of events** | **Single regression imputation** | **Multiple imputation** |
| --- | --- | --- | --- |
|  |  | **Βeta (95% CI)** | **Βeta (95% CI)** |
| No FT | 41 | Ref | Ref |
| Mild FT | 287 | -4.40 (-8.75, -0.06) | -4.40 (-8.76, -0.04) |
| Moderate FT | 230 | -8.67 (-13.22, -4.12) | -8.35 (-12.92, -3.78) |
| Severe FT | 14 | -18.09 (-26.29, -9.89) | -18.15 (-26.37, -9.93) |
| *p*-value (non-linearity) |  | 0.426 | 0.331 |
| Models are adjusted for age, center, religion, ethnicity, diagnosis, stage, diagnosis category, and treatment category | | | |

## References

[1] Buuren S van, Groothuis-Oudshoorn K. mice: Multivariate Imputation by Chained Equations in R. J Stat Softw 2011;45:1–67. https://doi.org/10.18637/jss.v045.i03.

[2] Multiple Imputation for Nonresponse in Surveys | Wiley Series in Probability and Statistics n.d. https://onlinelibrary.wiley.com/doi/book/10.1002/9780470316696 (accessed February 25, 2024).
